# Association of Health Literacy with the Implementation of Exercise during the Declaration of COVID-19 State of Emergency among Japanese Community-Dwelling Old-Old Adults

**DOI:** 10.3390/ijerph18042100

**Published:** 2021-02-21

**Authors:** Daijo Shiratsuchi, Hyuma Makizako, Yuki Nakai, Yoshiaki Taniguchi, Tomomi Akanuma, Kaori Yokoyama, Yuriko Matsuzaki-Kihara, Hiroto Yoshida

**Affiliations:** 1 Graduate School of Health Sciences, Kagoshima University, Kagoshima 890-8544, Japan; squall.lion8062@gmail.com (D.S.); p.taniguchi0601@gmail.com (Y.T.); 2 Department of Rehabilitation, Japan Community Health Care Organization Kumamoto General Hospital, Kumamoto 866-8660, Japan; 3 Department of Physical Therapy, School of Health Sciences, Faculty of Medicine, Kagoshima University, Kagoshima 890-8544, Japan; nakai@health.nop.kagoshima-u.ac.jp; 4 Department of Physical Therapy, Kagoshima Medical Professional College, Kagoshima 891-0133, Japan; 5 Bibai City Department of Health and Welfare Aged Care Division, Hokkaido 072-0026, Japan; t.akanuma@city.bibai.lg.jp (T.A.); k.yokoyama@city.bibai.lg.jp (K.Y.); 6 Rehabilitation Department, Health and Medical Faculty, Japan Health Care College, Hokkaido 004-0839, Japan; yuriko.mk@nihoniryo-c.ac.jp; 7 Graduate School of Health and Environment Science, Tohoku Bunka Gakuen University, Miyagi 981-8551, Japan; hiroto-y@hss.tbgu.ac.jp

**Keywords:** SARS-CoV-2, older person, health literacy, exercise

## Abstract

Health literacy is important for promoting and maintaining good health in old-old adults. It may influence the implementation of exercise in the coronavirus disease epidemic. The present cross-sectional study investigated the association of each dimension of health literacy with the implementation of exercise during the declaration of a state of emergency due to coronavirus disease in community-dwelling old-old adults. We collected data from 483 community-dwelling old-old adults (52.8% women) aged between 77 and 99 years who participated in a mail survey. Participants were divided into exercise or nonexercise groups based on the implementation of exercise during the declaration of a state of emergency. Health literacy was assessed using a 14-item health literacy scale. There were 327 (67.7%) participants in the exercise group and 156 (32.3%) in the nonexercise group. A significantly higher score of health literacy was observed in the exercise group than in the nonexercise group (communicative health literacy score = 14.0 ± 3.6 vs. 12.7 ± 3.8, *p* = 0.001). In a multivariate logistic regression model adjusted for potential confounders, high communicative health literacy scores were significantly associated with the implementation of exercise during the declaration of a state of emergency (odds ratio = 1.88, 95% confidence interval = 1.20–2.93). Approximately two-thirds of community-dwelling old-old adults implement exercise during the declaration of a state of emergency. High communicative health literacy was associated with the implementation of exercise during this period.

## 1. Introduction

As of January 2021, coronavirus disease (COVID-19), which has so far had a major impact on the medical systems and economies in the world, has infected about 88 million people worldwide and killed about 1.9 million people [1]. Old-old adults with reduced resilience are at high risk of death from COVID-19 [2,3], and there are concerns that the risk of long-term care will increase due to restrictions on going out, such as declarations of emergencies. The declaration of a state of emergency in Japan, which had no legal force and required people to refrain from going outside for unnecessary and nonurgent tasks, was implemented from April 7 to May 25. Japan’s population aging rate is one of the highest in the world, exceeding 28% as of 2018. As for the breakdown of the aging rate, 13.9% were older adults aged 65 to 74 years, 14.2% were older adults aged 75 years and older, and more than half were old-old adults [4]. The term “old-old adults” means older adults aged 75 years and above. Old-old adults need to exercise more actively than in normal times to maintain their health during an infectious disease epidemic.

In previous studies, physical activity is associated with age, sex, health status, self-efficacy, motivation, and health literacy (HL) [5,6]. HL is an individual’s ability and skill to access, understand, appraise, and apply health information in ways that promote and maintain good health during their life course [7]. Nutbeam divides HL into three constructs, each advocating functional, communicative, and critical HL. Functional HL refers to basic literacy, while communicative or critical HL is defined as more advanced literacy skills. Specifically, functional HL refers to basic skills of reading and writing and the ability to function effectively in everyday situations. Communicative HL, together with social skills, can be used to actively participate in every activity, extract information and derive meaning from different forms of communication, and apply new information to changing circumstances. Critical HL can be applied to critically analyze information and to use this information to exert greater control over life events and situations [8]. It has been clarified that HL decreases with aging [9], and it is considered to be one of the skills to be evaluated in the field of care prevention in developed countries where aging is progressing.

It has been suggested that low HL may accelerate the future decline of physical and cognitive function as well as increase mortality [10,11,12]. Low HL, although in cross-sectional observational studies, has also been found to be associated with a lack of exercise habits and frailty [13,14]. In addition, a report examining the three components of HL found that communicative HL was most strongly associated with the patients’ perceived ability to organize care [15].

Even under the COVID-19 epidemic, HL may affect the health behavior of old-old adults, especially the implementation of physical exercise, and maintain good health, but there are no reports on this. In addition, there are no reports that have examined whether communicative or critical HL is more associated with exercise implementation. Thus, this study aimed to investigate the relationship between HL and the implementation of exercise during the declaration of emergency in old-old adults living in the community. Clarifying the effects of HL on exercise behavior under the COVID-19 epidemic situation can be useful when supporting old-old adults in an infectious disease epidemic.

## 2. Materials and Methods

### 2.1. Population and Participants

This cross-sectional study was conducted by mailing a self-administered questionnaire in Bibai City, Hokkaido Government, Japan. A mail survey was conducted for 1112 community-dwelling older adults who participated in the health check program, which was conducted in 2018, and were aged 75 years and above. Bibai is a rural city with a population of 20,839 and an aging population of 42.5% in 2020 [16]. The declaration of a state of emergency in Bibai, which had no legal force and required people to refrain from going outside for unnecessary and nonurgent tasks, was implemented from 28 February to 19 March, 2020. The mail survey and collection of the questionnaire were conducted between 15 July and 16 September, 2020. Of the 1112 old-old adults, 889 responded to this survey. Respondents with a history of stroke, dementia, Parkinson’s disease, or depression were excluded from the analyses. Those who lacked each variable were also excluded. Finally, we analyzed the data of 483 respondents.

This study was conducted with the approval of the Ethics Committee of the Faculty of Medicine, Kagoshima University (No. 200065). Informed consent was obtained from all participants prior to their inclusion in the study, and sufficient ethical considerations were given based on the Declaration of Helsinki.

### 2.2. Physical Exercise Behavior

Regarding the implementation of physical exercise during the state of emergency, participants were asked to answer the following question, “What kind of exercise did you do during the state of emergency? Included even for a short time.” Participants selected multiple responses out of the five responses: walking, radio calisthenics, stretching, others, and none. Those who selected none were classified into the nonexercise group, and those who selected some exercise were classified into the exercise group. Radio calisthenics is a traditional form of exercise in Japan and has been shown to have a relatively high rate of implementation and effect on preventing the loss of skeletal muscle mass [17,18].

### 2.3. Health Literacy

The 14-item health literacy scale was used for HL evaluation [19,20]. We rephrased the questionnaire and asked respondents to answer it with relevance to COVID-19. The original version was validated and reliability verified for Japanese people, and Cronbach’s alpha was reported to be 0.76–0.85 [20]. The basic/communicative HL consists of 5 questions each, and the critical HL consists of 4 questions, for a total of 14 questions. From the fact that they strongly disagree or strongly agree, they get 1–5 points in 5 stages until they strongly agree, and the total score is 70 points, which means that the higher the score, the higher the HL. Japan has an educational history equal to or higher than that of European countries, and it is expected that the basic HL is high [21,22]. In this survey, we investigated eight items focusing on communicative/critical HL. Researchers have established the internal consistency of this scale, with a Cronbach’s alpha of 0.88. The specific question item was: “Since the declaration of a state of emergency in Hokkaido and you have little information about the COVID-19 and its treatment, how do you agree or disagree about the following?” (1) “I collect information from various sources,” (2) “I extract the information I want,” (3) “I understand the obtained information,” and (4) “I apply the obtained information to my daily life.” “Since the declaration of a state of emergency in Hokkaido and you can obtain information about the COVID-19 and its treatment, how do you agree or disagree about the following?” (5) “I consider whether the information is applicable to me,” (6) “I consider whether the information is credible,” (7) “I check whether the information is valid and reliable,” and (8) “I collect information to make my healthcare decisions.” The question “I tell my opinion about my illness to my doctor, family, or friends” was deleted in advance as it may bias the answer because this survey was conducted during the COVID-19 state of emergency. 

### 2.4. Covariates

The covariates were age, sex, presence or absence of cohabitants, 5-item frailty screening index, 5-item GDS, an outing to the town, and chronic pain. The 5-item frailty screening index was used to evaluate the frail state. The 5 items were as follows: (1) “Have you lost 2 kg or more in the past 6 months?” Yes = 1, (2) “Do you think you walk slower than before?” Yes = 1, (3) “Do you go for a walk for your health at least once a week” No = 1, (4) “Can you recall what happened 5 min ago?” No = 1, and (5) “In the past 2 weeks, have you felt tired without a reason?” Yes = 1. We defined scores of 3 or more as frail, 1 to 2 as prefrail, and 0 as robust [23]. The 5-item GDS was used to evaluate the depressive state [24]. The Cronbach’s alpha for the 5-item frailty screening index and 5-item GDS were 0.46 and 0.53, respectively. We also investigated whether or not they went out to the town at least once a week during the state of emergency and whether or not they had chronic low back pain and knee pain that lasted for 3 months or more. Chronic low back and knee pain were assessed through the following questions: “Do you have low back (knee) pain at the present time?” (yes or no). If yes, the following question was asked: “How long has the low back (knee) pain lasted?” (i) Less than 1 month, (ii) 1 to 3 months, and (iii) more than 3 months. Participants with either low back or knee pain, or both lasting more than 3 months, were categorized into the chronic pain group.

### 2.5. Statistical Analysis

The mean value ± standard deviation was calculated for continuous variables, and the count (%) was calculated for nominal variables. Student’s *t*-test for continuous variable and Pearson’s χ^2^-test for the nominal variable were used to examine the differences between the exercise and nonexercise groups. The association between HL and the implementation of physical exercise during the declaration of the COVID-19 state of emergency was examined using multivariate logistic regression analyses. The lower 25% of the HL score was defined as low health literacy. Although the time series of causality is reversed, HL in older adults is thought to change little in a short period of time [25,26]. The first model (Model 1) in the multivariate logistic regression analysis was adjusted for age, sex, and living alone. Model 2 included age, sex, living alone, frail, GDS-5, going out to the town, and the presence or absence of chronic pain as covariates. Adjusted odds ratios (OR) and 95% confidence intervals (95% CI) were calculated. All analyses were carried out using IBM SPSS Statistics 26.0 (IBM Japan, Tokyo, Japan). The level of statistical significance was set at *p* < 0.05.

## 3. Results

### 3.1. Subsection Characteristics of Participants

Table 1 shows a comparison of the characteristics between participants who exercised during the state of emergency and those who did not. Participants in the nonexercise group were older, had more severe frailty determinations, had higher total GDS-5 scores, were less likely to be out of town during the declaration of an emergency, and have chronic pain than those in the exercise group. Old-old adults exercising during the state of emergency had significantly higher communicative HL (*p* = 0.001) and critical HL (*p* = 0.021).

**Figure 1:**
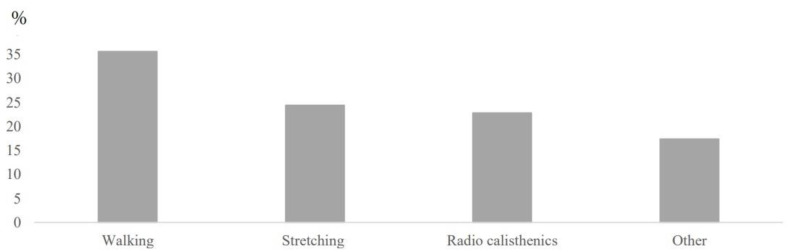
Assessment of the type of exercise during the COVID-19 pandemic state of emergency by the implementation of exercise during the declaration of a state of emergency containing one or more of the following was defined as the implementation of exercise: walking, radio calisthenics, stretching, other.

Figure 1 shows the results of the exercise types. Of all the participants, 327 (67.7%) performed some exercise during the state of emergency; 35.6% walked, 22.8% performed radio calisthenics, and 24.4% performed stretches. Other exercises were performed by 17.4% of the participants.

### 3.2. Association of Health Literacy with the Implementation of Exercise

Table 2 shows the results of the logistic regression analysis. The high total score of communicative HL was independently associated with performing some exercise during the state of emergency (OR = 1.98, 95% CI = 1.31–3.01; *p* = 0.001). This association remained significant after adjusting for age, sex, living alone, frailty, GDS-5 score, an outing to town, and chronic pain (OR = 1.88, 95% CI = 1.20–2.93; *p* = 0.006). Frailty and depression were also significantly associated with exercise during the COVID-19 state of emergency but not with critical HL.

## 4. Discussion

This study investigated the association of HL with the implementation of exercise during a state of emergency due to the COVID-19 epidemic among community-dwelling old-old adults. The results showed that high communicative HL was associated with the implementation of exercise during the declaration of the COVID-19 state of emergency even after adjusting for potential covariates. On the other hand, critical HL showed no significant association. To the best of our knowledge, this is the first study to show that HL is associated with the implementation of exercise during the declaration of COVID-19 state of emergency in community-dwelling old-old adults. Previous studies with community-dwelling old-old adults in normal times suggest that higher HL was associated with performing the exercise [13]. The results of the current study suggest that having a high HL was independently associated with physical exercise not only during normal times but also in emergencies such as infectious disease epidemics.

Given the cross-sectional design of this study, we were unable to conclude the causal relationship between HL and the performance of exercise, but HL may play a salient role in the progression of healthy behavior in this population. Previous studies have reported, “Improved capacity to act independently on knowledge, improved motivation and self-confidence” as an individual benefit due to high communicative HL and “Improved individual resilience to social and economic adversity” as an individual benefit due to high critical HL [8]. It is clear that motivation is related to the implementation of exercise [27]. This HL difference in effect may have influenced the implementation of the exercise during the declaration of the COVID-19 state of emergency. However, further research is needed. It has also been shown that Japanese people aged 20 to 64 years with high HL have a lower smoking rate and drinking frequency, and an increase in exercise behavior [28]. The need for exercise in maintaining the health of old-old adults is obvious and has been reported to improve physical function as well as the quality of life [29,30,31,32].

It has been suggested that infection control measures such as lockdown and emergency declaration associated with COVID-19 reduce the amount of physical activity including exercise [33,34]. Having a high HL may lead to the acquisition and implementation of more appropriate infection control and more efficient exercise information as the media disseminates various information in the COVID-19 state of emergency. Walking was the most common type of exercise performed during the declaration of the COVID-19 state of emergency, and older adults with high HL may have been able to perform their usual exercise during the declaration of a state of emergency after implementing infection control measures. Thus, HL should be paid more attention to in the maintenance and promotion of health in old-old adults.

In recent years, intervention studies have also been conducted to improve HL in older adults [26]. A previous study reported improvement in HL as a result of 24 weeks of active learning for community-dwelling older adults. The study also found that older adults in the intervention group took about 2400 more steps per day on average. Thus, for older people with low HL, raising the HL may help promote athletic behavior. In addition, people with high HL may want to try effective ways to further increase their athletic behavior, such as setting a step goal and the use of a step diary in that order [35]. The implementation of the exercise during the declaration of the COVID-19 emergency was also related to frailty status. Previous studies have shown that frailer individuals are more likely to spend more time on sedentary behavior [36]. Frailty in older adults may negatively impact their ability to exercise and increase the risk of adverse health effects. Although frailty screening and counteractive interventions are performed in clinical practice, they are not sufficiently developed yet [37]. Severe cases of frailty are less likely to benefit from exercise interventions; hence, early detection is necessary in such patients [38,39]. To prevent the transition to a state in need of care by not performing the exercise, they should be as physically active as their abilities and conditions allow [40].

Several limitations of this cross-sectional study should be noted. First, it was difficult to generalize the results of this survey due to regional differences in the status of COVID-19 infection and the timing of emergency declarations. The participants of this survey lived in a specific area of Hokkaido and were not randomly selected samples. Second, a low valid response rate may have resulted in selection bias. In addition, the impossibility of generalizing the data due to changes in the 14-item health literacy scale. Third, the sample size was not large. Fourth, we could not measure physical exercise behaviors using accelerometers or pedometers, they were assessed using a simple questionnaire rather than a validated index. Although the validity of such surveys is not well established, face-to-face diagnosis during the COVID-19 pandemic is difficult, and hence, questionnaire surveys are used because of their simplicity and ease of classification [41]. Finally, the effects of other unmeasured confounding factors, such as cognitive function, were not tested. Considering these limitations, the findings of the present study should be interpreted with caution.

## 5. Conclusions

In conclusion, HL was associated with the implementation of exercise during the state of emergency among community-dwelling old-old adults. Thus, HL could be an important factor in promoting exercise behavior even during a COVID-19 state of emergency.

## Figures and Tables

**Table 1:** Comparisons of characteristics between old-old adults who performed physical exercise and those who did not during the state of emergency due to the coronavirus disease (COVID-19) pandemic.

Variable	Total	Exercise	Nonexercise	*p* *
	(*n* = 483)	(*n* = 327)	(*n* = 156)	
Age, years	82.8 ± 4.3	82.4 ± 4.2	83.7 ± 4.5	0.002
Female, *n* (%)	256 (52.8%)	173 (52.9%)	82 (52.6%)	0.944
Living alone, *n* (%)	137 (28.4%)	97 (29.7%)	40 (25.6%)	0.359
Frail, *n* (%)				<0.001
Robust	65 (13.5%)	60 (18.3%)	5 (3.2%)	
Prefrail	310 (64.2%)	217 (66.4%)	93 (59.6%)	
Frail	108 (22.4%)	50 (15.3%)	58 (37.2%)	
GDS-5, score	1.24 ± 1.20	1.03 ± 1.00	1.68 ± 1.44	<0.001
Outing to the town during the state of emergency by the COVID-19 pandemic, *n* (%)	323 (66.9%)	229 (70.0%)	94 (60.3%)	0.033
Chronic low back pain or chronic knee pain, *n* (%)	242 (50.1%)	148 (45.3%)	94 (60.3%)	0.002
Communicative Health Literacy, score (range = 4–20)	13.6 ± 3.7	14.0 ± 3.6	12.7 ± 3.8	0.001
Critical Health Literacy, score (range = 4–20)	12.7 ± 3.7	12.9 ± 3.7	12.1 ± 3.5	0.021

Data are shown as the mean ± standard deviation or percentage. SD, standard deviation. * Student’s *t*-test for continuous measures and Pearson’s χ^2^-test for proportions.

**Table 2:** Associations of exercise during the COVID-19 pandemic state of emergency with health literacy.

	Exercise during the COVID-19 Pandemic State of Emergency			
	Model 1		Model 2	
Independent Variables	OR (95% CI)	*p*	OR (95% CI)	*p*
Communicative Health Literacy	1.98 (1.31–3.01)	0.001	1.88 (1.20–2.93)	0.006
Critical Health Literacy	1.05 (0.66–1.68)	0.828	0.96 (0.58–1.58)	0.868
Age	0.94 (0.90–0.98)	0.005	0.96 (0.92–1.01)	0.121
Female	0.97 (0.65–1.45)	0.884	1.15 (0.75–1.76)	0.532
Living alone	1.29 (0.82–2.03)	0.274	1.54 (0.94–2.52)	0.088
Frail				
Robust			1.000	
Prefrail			0.26 (0.10–0.69)	0.006
Frail			0.12 (0.04–0.34)	<0.001
GDS-5			0.76 (0.63–0.91)	0.002
Outing to the town			0.94 (0.60–1.47)	0.777
Chronic low back pain or chronic knee pain			1.44 (0.94–2.20)	0.098

Note: OR = odds ratio; CI = confidence interval. Model 1: Adjusted for age, sex, and living alone. Model 2: Adjusted for age, sex, living alone, frail, GDS-5, an outing to the town during the state of emergency by the COVID-19 pandemic, chronic low back pain, or chronic knee pain.

## Data Availability

Data sharing not applicable.
